# Prescribing trends of proton pump inhibitors, antipsychotics and benzodiazepines of medicare part d providers

**DOI:** 10.1186/s12877-022-02971-2

**Published:** 2022-04-09

**Authors:** Jennifer M. Toth, Saumil Jadhav, Holly M. Holmes, Manvi Sharma

**Affiliations:** 1 Department of Pharmacy Administration, The University of Mississippi, University, MS 38677 USA; 2 Division of Geriatric and Palliative Medicine, McGovern Medical School, The University of Texas Health Science Center, Houston, TX USA

**Keywords:** Medication use, Costs, Antipsychotic, Benzodiazepine, Proton pump inhibitor, Geriatric, Deprescribing

## Abstract

**Background:**

Proton pump inhibitors, benzodiazepines, and antipsychotics are considered potentially inappropriate medications in older adults according to the American Geriatric Society Beers Criteria, and deprescribing algorithms have been developed to guide use of these drug classes. The objective of this study was to describe the number of beneficiaries prescribed these medications, provider specialty and regional trends in prescribing, and the aggregate costs for these claims in Medicare Part D.

**Methods:**

This was a retrospective cross-sectional study using publicly available Medicare Provider Utilization and Payment Data: Part D Prescriber data for years 2013–2019. Descriptive statistics and the Cochrane-Armitage test were used to summarize the trends.

**Results:**

Overall, 30.1%, 25.6%, 4.6% of Medicare Part D beneficiaries had a proton pump inhibitor, benzodiazepine, and antipsychotic claim in 2013, respectively. These rates decreased to 27.5%, 17.5%, 4.1% in 2019 (*p*-value < 0.0001). However, the number of standardized 30-day claims increased from 63 million in 2013 to 84 million in 2019 for proton pump inhibitors, remained steady for benzodiazepines and slightly increased (10 million to 13 million) for antipsychotics. Total aggregate costs decreased by almost $1.5 billion for proton pump inhibitor, $100 million for benzodiazepine, and $700 million for antipsychotic from 2013 to 2019 (*p*-value < 0.0001). Almost 93% of gastroenterologists prescribed a proton pump inhibitor, and 60% of psychiatrists prescribed benzodiazepines and antipsychotics all seven years. The Other region had the highest percentage of providers prescribing all three classes and the highest number of standardized 30-day benzodiazepine claims.

**Conclusions:**

The overall rate of use of proton pump inhibitors, benzodiazepines, and antipsychotics decreased from 2013–2019 among Medicare Part D beneficiaries. Despite the increase in raw number of standardized 30-day claims, the costs decreased which is likely due to generics made available. These prescribing trends may aid in identifying and targeting potential deprescribing interventions.

## Introduction

The American Geriatrics Society Beers criteria includes proton pump inhibitors (PPIs), antipsychotics (APs) and benzodiazepines, including z-sleep aids, (BZRAs) as potentially inappropriate drug classes for older adults [1]. Deprescribing algorithms have been developed for PPIs, APs and BZRAs to guide safe discontinuation of these classes of drugs when indicated or warranted [2–4]. Deprescribing has been defined as the “systematic process of identifying and discontinuing drugs when existing or potential harms outweigh existing or potential benefits within the context of an individual patient’s care goals, functional status, life expectancy, values, and preferences” [5]. After at least four weeks of PPI treatment and resolution of gastrointestinal symptoms, there is a strong recommendation to stop or decrease the dose of the PPI or use it as needed with a few exceptions [2]. APs should be stopped for insomnia and tapered for dementia symptoms that are controlled and have been treated for more than three months [3]. BZRAs are strongly recommended to be slowly tapered in elderly adults who are using these for insomnia [4]. These medications are recommended for deprescribing because benefits, if any, do not outweigh the risks.

Overall, use of these medications needs to be individualized with consideration of each patient’s condition being treated, concomitant medications, and comorbidities. A review suggests using PPIs for the shortest time possible favoring conditions that would benefit the most from PPIs [6]. A meta-analysis that compared the effectiveness and adverse effects of specific APs concluded benefits and risks of using antipsychotics need to be assessed in each patient [7].

As with any drug, there are benefits as well as safety concerns. However, older adults may be more prone to these adverse effects. A few common adverse effects of PPIs found in the older adult population from claims data include increased risk for *Clostridium difficile *infections, bone fractures, and myocardial infarctions if on concomitant clopidogrel [8–13]. Recently, current PPI use was found to be associated with worse COVID-19 outcomes in a Korean sample [14]. The benefits of PPIs outweigh the risks when indicated, but the need for PPIs should be assessed at subsequent health care visits [15]. Reduced mortality and hospitalization from upper gastrointestinal bleeds with concomitant non-steroidal anti-inflammatory drugs, oral anticoagulation, COX-2 inhibitors, or salicylates are reported benefits of PPIs from claims data [16–19]. Multiple adverse effects, including increased risk of mortality, stroke, extrapyramidal symptoms, and pneumonia are associated with some or all APs [20]. APs have not been found to help prevent or treat delirium in inpatients and are associated with falls [21, 22]. BZRAs have been associated with falls, dependence, decrease in cognitive function, and mortality [22, 23].

Each of these three drug classes is frequently inappropriately prescribed in older adults, despite the adverse benefit/risk profile. PPIs are a highly prescribed medication. In a study of National Ambulatory Medical Care Survey data from 2006–10, PPI prescriptions were issued during 329 million estimated outpatient visits [24]. Over 50–80% of patients discharged from the hospital have been found to be inappropriately prescribed a PPI [25–27]. Continuing the PPI 30 days post-discharge cost a managed care organization more than $3 million over four years [27]. In residential facility patients with dementia, around 30% used an AP every year, and 65% of these users were on an AP for longer than three months [28]. In 2008, 8.7% of US adults 65–80 years old used BZRAs, and 2.7% used BZRAs long-term [29]. About 12% of Norwegians who used BZRAs aged 70–89 years were found to not use them appropriately [30].

Because these medications are recommended to be used not at all, with caution, or for a short duration in the older adult population, identifying prescribing trends can highlight where deprescribing efforts should be targeted. The objectives of this research were to describe the prescribing trends and prevalence, per physician specialty type and US region and to estimate the aggregate costs of PPIs, APs and BZRAs based on Medicare Part D claims in beneficiaries ≥ 65 years.

## Methods

### Design

This is a retrospective, observational study that aims to characterize prescribing trends of PPIs, APs, and BZRAs in the Medicare Part D population over the age of 65. This study has been approved as exempt by the University of Mississippi Institutional Review Board.

### Data sources

Data for this study came from Medicare Provider Utilization and Payment Data: Part D Prescriber for calendar years 2013–19, which are publicly available from Centers for Medicare & Medicaid Services [31]. The Detailed Data, Provider Summary Table, and National Drug Summary Table for each year were utilized for 2013–18 data, and the Medicare Part D prescribers – by geography and drug, Medicare Part D prescribers—by provider, and Medicare Part D Prescribers—by provider and drug sets were used for 2019 data. The Detailed Dataset and Medicare Part D prescribers—by provider and drug allowed for analysis of providers and the count of unique beneficiaries to whom they prescribed PPIs, APs, or BZRAs each year. The Provider Summary Table and Medicare Part D Prescribers—by provider were used for gathering characteristics on prescribers. Overall prescribing prevalence for individual and total drug use were calculated from the National Drug Summary Table and Medicare Part D prescribers – by geography and drug for each year. Total Medicare Part D beneficiaries were found in Medicare Part D grand totals report. Aggregated values from 10 or less beneficiaries are suppressed [32].

### Participants

The study population of interest was Medicare Part D beneficiaries ≥ 65 years and their providers. Beneficiaries over the age of 65 years constitute most of the Medicare Part D population at about 83%. Counts of beneficiaries ≥ 65 years are already separated in the data sets and are identified by “GE65_” at the beginning of the variable name or “_65” at the end of the variable name. Data from beneficiaries who used PPIs, APs, or BZRAs were included.

Using the National Drug Summary Table and Medicare Part D prescribers – by geography and drug, PPIs were found by searching by generic name, including dexlansoprazole, esomeprazole, lansoprazole, omeprazole, pantoprazole, and rabeprazole. PPIs that were in intravenous form were excluded from the study. APs were searched by name, and included amoxapine, aripiprazole, asenapine, chlorpromazine, clozapine, haloperidol, iloperidone, loxapine, olanzapine, paliperidone, quetiapine, and risperidone. BZRAs were found by generic names, including alprazolam, chlordiazepoxide, clonazepam, clorazepate, diazepam, estazolam, eszopiclone, flurazepam, lorazepam, oxazepam, temazepam, triazolam, and zolpidem. Midazolam and diazepam rectal gel were excluded.

### Variables

Prevalence of use of each drug was calculated by summing the unique beneficiaries over the age of 65 years (unique_bene_65 or GE65_Tot_Benes) that were prescribed each drug and divided by the total number of Medicare Part D beneficiaries over the age of 65 years for each year. Total standardized 30-day claims and total aggregated costs for each drug were calculated by summing their respective variables. Standardized 30-day claim was defined in the data set as the number of days’ supply on a claim divided by 30, and total aggregated costs include all costs associated with the drug claim [33].

Similarly, prescribers who prescribed each drug class were found on the Detailed Data and Medicare Part D prescribers – by provider and drug set and matched with their National Provider Identifier on the Provider Summary Table and Medicare Part D prescribers—by provider. Prescribers were categorized in five broader specialty categories based on their specialty. These specialties include surgeons, gastroenterology, general practitioners, mid-level practitioners, and alternative medicine practitioners for PPIs. Geriatric medicine physicians were included in general practitioners. For APs and BZRAs specialties were categorized as surgeons, sleep medicine, psychiatrist, general practitioner, mid-level practitioners, and alternative medicine practitioners. Geriatric psychiatrists were included with psychiatrists. General practitioners included internal medicine, family medicine, and unknown providers. Mid-level practitioners included non-physician health providers. Prescribers were categorized into five regions, Northeast, Midwest, South, West, Other, based on their state or territory [34]. The Other region includes U.S. territories, armed forces located outside the U.S., foreign countries, and unknown areas. Total number of prescribers of each drug class and standardized 30-day claims for beneficiaries over 65 years were calculated for each specialty and region. Counts from providers that have prescribed certain medications to < 11 beneficiaries were suppressed, which could lead to lower values than what would be found in the true population.

### Statistical analysis

SAS version 9.4 (Cary, NC) was used to analyze the data. Descriptive statistics were used to summarize the number of beneficiaries over 65 years with a claim for each drug, number of standardized 30-day claims for beneficiaries over 65 years, aggregate costs, and number of prescribers and standardized 30-day claims for beneficiaries over 65 years grouping by specialty and region. To test the significance of temporal trends, the percentage change in the proportion of use over time for totals were estimated with use of the Cochran–Armitage trend test. Sensitivity of analyses of trend tests were done with logistic regression using the log-likelihood ratio and the Wald test. All tests were two-sided with an alpha of 0.05.

## Results

### PPIs

The overall prevalence of PPI prescriptions in 2013 for the Medicare Part D population over the age 65 years was 30.1%. The prevalence increased to 30.3% in 2015 and decreased to 27.5% in 2019 (*p* < 0.0001). Omeprazole was the drug with the highest prevalence, which decreased from 17.7% in 2013 to 14.7% in 2019. The prevalence of pantoprazole prescriptions increased from 6.1% in 2013 to 9.8% in 2019. The number of beneficiaries and prevalence of beneficiaries over the age of 65 years prescribed a PPI from 2013 to 2019 for each PPI can be seen in Table 1.

**Table 1:** Number of beneficiaries over the age of 65 years with at least one claim for a PPI, benzodiazepine, or antipsychotic from 2013 to 2019^a^

PPI Class	2013 (*n* = 28,844,629)	2014 (*n* = 30,684,148)	2015 (*n* = 32,313,123)	2016 (*n* = 33,891,805)	2017 (*n* = 35,372,145)	2018 (*n* = 36,932,602)	2019 (*n* = 38,553,139)
Dexlansoprazole	226,986 (0.8%)	225,310 (0.7%)	232,998 (0.7%)	233,815 (0.7%)	223,256 (0.6%)	211,981 (0.6%)	201,243 (0.5%)
Esomeprazole	1,111,550 (3.9%)	1,061,160 (3.5%)	1,076,591 (3.3%)	916,632 (2.7%)	797,721 (2.3%)	688,701 (1.9%)	672,584 (1.7%)
Lansoprazole	387,702 (1.3%)	380,258 (1.2%)	364,983 (1.1%)	300,835 (0.9%)	244,572 (0.7%)	225,696 (0.6%)	230,987 (0.6%)
Omeprazole	5,108,489 (17.7%)	5,395,876 (17.6%)	5,599,523 (17.3%)	5,671,998 (16.7%)	5,588,075 (15.8%)	5,555,228 (15.0%)	5,649,420 (14.7%)
Pantoprazole	1,758,007 (6.1%)	2,146,790 (7.0%)	2,450,771 (7.6%)	2,738,089 (8.1%)	3,076,216 (8.7%)	3,407,091 (9.2%)	3,781,609 (9.8%)
Rabeprazole	75,443 (0.3%)	61,212 (0.2%)	54,968 (0.2%)	52,534 (0.2%)	49,292 (0.1%)	48,827 (0.1%)	50,459 (0.1%)
Total^b^	8,668,177 (30.1%)	9,270,606 (30.2%)	9,779,834 (30.3%)	9,913,903 (29.3%)	9,979,132 (28.2%)	10,137,524 (27.4%)	10,586,302 (27.5%)
Benzodiazepines							
Alprazolam	1,786,661 (6.2%)	1,865,302 (6.1%)	1,980,676 (6.1%)	2,009,077 (5.9%)	2,000,549 (5.7%)	1,965,284 (5.3%)	1,905,324 (4.9%)
Chlordiazepoxide	31,937 (0.1%)	27,715 (< 0.1%)	25,178 (< 0.1%)	29,595 (< 0.1%)	30,014 (< 0.1%)	26,637 (< 0.1%)	25,674 (< 0.1%)
Clonazepam	752,162 (2.6%)	813,472 (2.7%)	844,327 (2.6%)	871,342 (2.6%)	886,423 (2.5%)	891,142 (2.4%)	878,711 (2.3%)
Clorazepate	55,888 (0.2%)	57,288 (0.2%)	54,743 (0.2%)	50,707 (0.1%)	46,122 (0.1%)	40,986 (0.1%)	34,705 (< 0.1%)
Diazepam	664,643 (2.3%)	684,480 (2.2%)	679,446 (2.1%)	694,963 (2.1%)	697,061 (2.0%)	687,401 (1.9%)	671,488 (1.7%)
Estazolam	10,435 (< 0.1%)	9439 (< 0.1%)	9190 (< 0.1%)	8651 (< 0.1%)	8307 (< 0.1%)	8300 (< 0.1%)	8567 (< 0.1%)
Eszopiclone	105,064 (0.4%)	139,817 (0.5%)	85,005 (0.3%)	76,631 (0.2%)	78,527 (0.2%)	81,222 (0.2%)	79,400 (0.2%)
Flurazepam	14,508 (< 0.1%)	10,050 (< 0.1%)	8148 (< 0.1%)	6463 (< 0.1%)	5075 (< 0.1%)	4731 (< 0.1%)	3359 (< 0.1%)
Lorazepam	1,617,143 (5.6%)	1,642,220 (5.4%)	1,700,107 (5.3%)	1,709,125 (5.0%)	1,667,926 (4.7%)	1,595,208 (4.3%)	1,531,912 (4.0%)
Oxazepam	21,148 (< 0.1%)	18,241 (< 0.1%)	15,736 (< 0.1%)	13,899 (< 0.1%)	12,134 (< 0.1%)	10,866 (< 0.1%)	9482 (< 0.1%)
Temazepam	524,470 (1.8%)	543,168 (1.7%)	557,689 (1.7%)	523,224 (1.5%)	480,709 (1.4%)	462,935 (1.3%)	419,709 (1.1%)
Triazolam	48,416 (0.2%)	35,747 (0.1%)	32,590 (0.1%)	28,584 (< 0.1%)	26,591 (< 0.1%)	26,758 (< 0.1%)	27,611 (< 0.1%)
Zolpidem	1,758,215 (6.1%)	1,608,998 (5.2%)	1,449,947 (4.5%)	1,288,142 (3.8%)	1,202,397 (3.4%)	1,185,920 (3.2%)	1,140,302 (3.0%)
Total^b^	7,390,690 (25.6%)	7,455,937 (24.3%)	7,442,782 (23.0%)	7,310,403 (21.6%)	7,141,835 (20.2%)	6,987,390 (18.9%)	6,736,244 (17.5%)
Antipsychotics							
Amoxapine	1555 (< 0.1%)	1527 (< 0.1%)	1439 (< 0.1%)	1383 (< 0.1%)	1250 (< 0.1%)	611(< 0.1%)	569 (< 0.1%)
Aripiprazole	126,298 (0.4%)	131,256 (0.4%)	196,453 (0.6%)	155,139 (0.5%)	165,097 (0.5%)	187,750 (0.5%)	213,379 (0.6%)
Asenapine	3586 (< 0.1%)	3620 (< 0.1%)	3636 (< 0.1%)	3639 (< 0.1%)	3388 (< 0.1%)	3220 (< 0.1%)	3141 (< 0.1%)
Chlorpromazine	21,011 (< 0.1%)	20,943 (< 0.1%)	20,062 (< 0.1%)	19,113 (< 0.1%)	18,950 (< 0.1%)	18,362 (< 0.1%)	17,757 (< 0.1%)
Clozapine	8,813 (< 0.1%)	9,362 (< 0.1%)	10,123 (< 0.1%)	10,730 (< 0.1%)	11,464 (< 0.1%)	12,278 (< 0.1%)	13,004 (< 0.1%)
Haloperidol	98,486 (0.3%)	94,136 (0.3%)	89,010 (0.3%)	83,392 (0.2%)	78,850 (0.2%)	72,109 (0.2%)	46.401 (0.1%)
Iloperidone	1337 (< 0.1%)	1329 (< 0.1%)	1183 (< 0.1%)	976 (< 0.1%)	989 (< 0.1%)	1039 (< 0.1%)	1011 (< 0.1%)
Loxapine	3214 (< 0.1%)	3341 (< 0.1%)	3277 (< 0.1%)	3318 (< 0.1%)	3295 (< 0.1%)	3348 (< 0.1%)	3477 (< 0.1%)
Olanzapine	191,485 (0.7%)	196,381 (0.6%)	203,320 (0.6%)	211,627 (0.6%)	222,829 (0.6%)	235,154 (0.6%)	248,824 (0.6%)
Paliperidone	4674 (< 0.1%)	5226 (< 0.1%)	7307 (< 0.1%)	7099 (< 0.1%)	7107 (< 0.1%)	7735 (< 0.1%)	8396 (< 0.1%)
Quetiapine	538,274 (1.9%)	572,652 (1.9%)	600,267 (1.9%)	631,804 (1.9%)	667,785 (1.9%)	705,936 (1.9%)	743,744 (1.9%)
Risperidone	339,875 (1.2%)	326,341 (1.1%)	315,664 (1.0%)	305,413 (0.9%)	296,788 (0.8%)	293,081 (0.8%)	291,000 (0.8%)
Total^b^	1,338,608 (4.6%)	1,366,114 (4.5%)	1,451,741 (4.5%)	1,433,633 (4.2%)	1,477,792 (4.2%)	1,540,623 (4.2%)	1,590,703 (4.1%)

^a^Percent of all Medicare Part D beneficiaries over the age of 65 years with a PPI, benzodiazepine, or antipsychotic claim from 2013 to 2018 in parentheses ^b^Per Cochran-Armitage trend test, *p*-value was < 0.0001 for all class level yearly trends

Table 2 shows the total number of standardized 30-day PPI claims for beneficiaries over the age of 65 years for each PPI from 2013–19. The number of standardized 30-day claims increased from 2013 to 2019. However, the number of claims per 1,000,000 standardized 30-day claims has slightly decreased from 41,604.5 to 39,660.6 (*p* < 0.0001). Pantoprazole was the only PPI whose number of claims per 1,000,000 increased from 7,638.2 per 1,000,000 in 2013 to 13,466.8 per 1,000,000 in 2019. However, omeprazole remained the most highly prescribed PPI despite its decrease in rate of claims (25,625.3 to 21,863.8 per 1,000,000 claims from 2013 to 2019).

**Table 2:** Standardized 30-day claims of all PPI, benzodiazepine, or antipsychotic drugs from 2013 to 2019 for beneficiaries over 65 years^a^^,b^

PPI Class	2013 (*n* = 1,525,617,121)	2014 (*n* = 1,623,590,539)	2015 (*n* = 1,705,969,287)	2016 (*n* = 1,807,356,444)	2017 (*n* = 1,900,796,290)	2018 (*n* = 2,002,880,967)	2019 (*n* = 2,107,865,407)
Dexlansoprazole	1,380,477 (904.9)	1,505,394 (927.2)	1,625,004 (952.5)	1,703,889 (942.8)	1,695,956 (892.2)	1,610,572 (804.1)	1,610,456 (764.0)
Esomeprazole	8,399,067 (5505.4)	7,893,796 (4861.9)	6,715,893 (3936.7)	6,098,071 (3374.0)	5,556,476 (2923.2)	5,242,718 (2617.6)	5,350,628 (2538.4)
Lansoprazole	2,544,238 (1667.7)	2,574,546 (1585.7)	2,599,759 (1523.9)	2,094,272 (1158.7)	1,828,125 (961.8)	1,706,397 (852.0)	1,758,957 (834.5)
Omeprazole	39,094,935 (25,625.7)	41,844,057 (25,772.5)	43,825,968 (25,689.8)	44,688,976 (24,726.2)	44,796,720 (23,567.3)	44,997,662 (22,466.5)	46,085,910 (21,863.8)
Pantoprazole	11,652,900 (7638.2)	14,646,066 (9020.8)	17,225,462 (10,097.2)	19,669,593 (10,883.1)	22,436,785 (11,803.9)	25,311,162 (12,637.4)	28,386,189 (13,466.8)
Rabeprazole	400,978 (262.8)	418,531 (257.8)	425,075 (249.2)	429,043 (237.4)	404,367 (212.7)	394,507 (197.0)	407,139 (193.2)
Total^c^	63,472,595 (41,604.5)	68,882,390 (42,426.0)	72,417,161 (42,449.3)	74,683,844 (41,322.1)	76,718,429 (40,361.2)	79,263,017 (39,574.5)	83,599,279 (39,660.6)
Benzodiazepines							
Alprazolam	9,054,121 (5934.7)	9,984,091 (6149.4)	10,647,341 (6241.2)	10,922,480 (6043.3)	10,791,550 (5677.4)	10,562,729 (5273.8)	10,065,051 (4775.0)
Chlordiazepoxide	133,488 (87.5)	137,074 (84.4)	125,893 (73.8)	138,132 (76.4)	143,115 (75.3)	129,533 (64.7)	123,152 (58.4)
Clonazepam	4,931,284 (3232.3)	5,554,184 (3420.9)	5,845,004 (3426.2)	6,117,389 (3384.7)	6,214,469 (3269.4)	6,236,771 (3113.9)	6,122,109 (2904.4)
Clorazepate	300,691 (197.1)	329,567 (203)	322,971 (189.3)	301,343 (166.7)	272,138 (143.2)	242,187 (120.9)	200,841 (95.3)
Diazepam	2,245,451 (1471.8)	2,443,486 (1505.0)	2,462,614 (1443.5)	2,505,014 (1386.0)	2,457,803 (1293.0)	2,351,823 (1174.2)	2,237,533 (1061.5)
Estazolam	45,475 (29.8)	47,633 (29.3)	46,411 (27.2)	46,938 (26)	46,512 (24.5)	45,366 (22.7)	49,049 (23.3)
Eszopiclone	580,254 (380.3)	526,896 (324.5)	419,267 (245.8)	408,970 (226.3)	447,786 (235.6)	463,569 (231.5)	466,642 (221.4)
Flurazepam	50,117 (32.9)	41,737 (25.7)	36,383 (21.3)	28,040 (15.5)	22,756 (12)	23,313 (11.6)	9776 (4.6)
Lorazepam	7,761,603 (5087.5)	8,265,974 (5091.2)	8,551,799 (5012.9)	8,687,678 (4806.8)	8,362,089 (4399.3)	7,919,633 (3954.1)	7,433,428 (3536.5)
Oxazepam	117,709 (77.2)	114,033 (70.2)	103,174 (60.5)	91,252 (50.5)	79,027 (41.6)	71,611 (35.8)	62,830 (29.8)
Temazepam	2,791,499 (1829.8)	3,155,738 (1943.7)	3,162,469 (1853.8)	2,985,863 (1652.1)	2,828,484 (1488.1)	2,782,561 (1389.3)	2,460,749 (1167.4)
Triazolam	167,771 (110)	154,825 (95.4)	142,649 (83.6)	120,877 (66.9)	112,632 (59.3)	107,776 (53.8)	103,389 (49.0)
Zolpidem	9,604,632 (6295.6)	8,214,618 (5059.5)	6,626,870 (3884.5)	5,952,027 (3293.2)	6,319,613 (3324.7)	6,658,375 (3324.4)	6,550,034 (3107.4)
Total^c^	37,784,095 (24,766.4)	38,969,856 (24,002.3)	38,492,846 (22,563.6)	38,306,005 (21,194.5)	38,097,973 (20,043.2)	37,595,246 (18,770.6)	35,884,583 (17,024.1)
Antipsychotics							
Amoxapine	14,580 (9.6)	14,304 (8.8)	13,658 (8.0)	13,022 (7.2)	10,523 (5.5)	3548 (1.8)	4527 (2.1)
Aripiprazole	1,039,944 (681.7)	1,097,009 (675.7)	1,061,993 (622.5)	1,107,915 (613.0)	1,261,167 (663.5)	1,453,819 (725.9)	1,680,325 (797.2)
Asenapine	22,847 (15.0)	24,668 (15.2)	25,991 (15.2)	27,770 (15.4)	28,319 (14.9)	28,525 (14.2)	28,477 (13.5)
Chlorpromazine	96,755 (63.4)	95,848 (59.0)	91,879 (53.9)	87,516 (48.4)	84,630 (44.5)	84,354 (42.1)	87,195 (41.4)
Clozapine	165,723 (108.6)	177,990 (109.6)	187,585 (110.0)	198,042 (109.6)	205,617 (108.2)	221,301 (110.5)	229,311 (108.8)
Haloperidol	512,097 (335.7)	503,232 (310.0)	484,145 (283.8)	471,944 (261.1)	454,201 (239.0)	418,037 (208.7)	254,519 (120.7)
Iloperidone	11,416 (7.5)	13,468 (8.3)	12,824 (7.5)	11,546 (6.4)	11,145 (5.9)	11,922 (6.0)	12,142 (5.8)
Loxapine	30,034 (19.7)	31,148 (19.2)	31,149 (18.3)	34,058 (17.7)	32,667 (17.2)	33,333 (16.6)	32,799 (15.6)
Olanzapine	1,510,182 (989.9)	1,566,810 (965.0)	1,632,941 (957.2)	1,730,381 (957.4)	1,822,656 (958.9)	1,924,529 (960.9)	2,030,909 (963.5)
Paliperidone	45,177 (29.6)	50,295 (31.0)	53,023 (31.1)	58,124 (32.2)	63,752 (33.5)	71,569 (35.7)	79,408 (37.7)
Quetiapine	4,303,901 (2821.1)	4,605,672 (2836.7)	4,879,421 (2860.2)	5,213,015 (2884.3)	5,569,245 (2930.0)	5,978,830 (2985.1)	6,409,240 (3040.6)
Risperidone	2,617,402 (1715.6)	2,562,152 (1578.1)	2,497,330 (1463.9)	2,488,164 (1376.7)	2,461,674 (1295.1)	2,468,146 (1232.3)	2,511,367 (1191.4)
Total^c^	10,370,056 (6797.3)	10,742,595 (6616.6)	10,971,938 (6431.5)	11,439,496 (6329.4)	12,005,595 (6316.1)	12,697,912 (6339.8)	13,360,219 (6338.3)

^a^Total may be more or less than sum of columns due to rounding ^b^Standardized 30-day PPI, benzodiazepine, or antipsychotic claims per 1,000,000 30-day claims in parentheses ^c^Per Cochran-Armitage trend test, *p*-value was < 0.0001 for all total class level yearly trends

Total aggregate costs per $1,000,000 for PPIs decreased from $43,282 in 2013 to $12,575 in 2019 (*p* < 0.0001). Costs for dexlansoprazole and pantoprazole increased. All others had decreasing costs. Dexlansoprazole was the only PPI that had increasing costs per $1,000,000 from $3,016 in 2013 to $3,989 in 2017, which then decreased to $3437 in 2019. Other aggregates costs are in Table 3.

**Table 3:** Aggregate Cost in U.S dollars of all PPI, benzodiazepine, or antipsychotic drugs from 2013 to 2019 for beneficiaries over 65 years^a^^,b^

PPI Class	2013 (*n* = $71,050,836,274)	2014 (*n* = $81,973,711,182)	2015 (*n* = $92,529,508,750)	2016 (*n* = $100,249,753,916)	2017 (*n* = $107,531,371,999)	2018 (*n* = $119,157,323,622)	2019 (*n* = $132,354,368,318)
Dexlansoprazole	214,257,666 (3016)	271,867,604 (3317)	345,694,553 (3736)	400,355,153 (3994)	428,920,381 (3989)	440,172,126 (3694)	454,994,317 (3438)
Esomeprazole	1,838,406,947 (25,875)	1,950,215,744 (23,791)	1,656,840,791 (17,906)	1,160,036,693 (11,571)	790,467,867 (7351)	548,797,134 (4606)	392,139,694 (2963)
Lansoprazole	175,009,663 (2463)	145,050,920 (1769)	124,945,073 (1350)	89,526,564 (893)	70,548,353 (656)	66,721,947 (560)	61,824,846 (467)
Omeprazole	546,017,766 (7685)	458,143,392 (5589)	402,350,325 (4348)	430,758,245 (4297)	465,339,922 (4327)	461,354,660 (3872)	463,478,280 (3502)
Pantoprazole	169,448,043 (2385)	171,077,701 (2087)	173,546,791 (1876)	187,518,248 (1871)	204,724,125 (1904)	221,413,798 (1858)	260,890,013 (1971)
Rabeprazole	132,084,336 (1859)	55,994,036 (683)	44,772,854 (484)	38,616,122 (385)	33,881,913 (315)	32,600,354 (274)	31,043,886 (235)
Total^c^	3,075,224,421 (43,282)	3,052,349,399 (37,236)	2,748,150,386 (29,700)	2,306,811,025 (23,011)	1,993,882,562 (18,542)	1,771,060,019 (14,863)	1,664,371,037 (12,575)
Benzodiazepines							
Alprazolam	59,779,546 (841)	64,897,520 (792)	70,841,054 (766)	78,206,882 (780)	76,334,553 (710)	82,787,025 (695)	78,495,459 (593)
Chlordiazepoxide	2,511,327 (35)	2,629,713 (32)	2,358,428 (25)	5,307,039 (53)	10,589,236 (98)	10,156,464 (85)	8,977,263 (68)
Clonazepam	36,337,909 (511)	36,943,872 (451)	35,498,553 (384)	46,603,866 (465)	42,401,690 (394)	44,309,680 (372)	45,475,579 (344)
Clorazepate	5,188,731 (73)	4,921,226 (60)	6,211,661 (67)	13,905,167 (139)	20,277,380 (189)	19,873,626 (167)	17,634,246 (133)
Diazepam	14,719,360 (207)	15,646,497 (191)	16,302,551 (176)	17,573,465 (175)	17,732,681 (165)	18,552,089 (156)	16,993,123 (128)
Estazolam	588,400 (8)	736,312 (9)	836,528 (9)	930,009 (9)	1,006,178 (9)	1,204,282 (10)	1,555,074 (12)
Eszopiclone	139,101,466 (1958)	101,569,293 (1239)	30,142,622 (326)	19,652,066 (196)	17,327,812 (161)	19,218,936 (161)	18,502,479 (138)
Flurazepam	314,929 (4)	353,429 (4)	465,248 (5)	488,256 (5)	442,444 (4)	463,938 (4)	185,259 (1)
Lorazepam	67,972,421 (957)	68,590,970 (837)	77,512,533 (838)	80,791,330 (806)	71,867,245 (668)	70,564,757 (592)	69,433,942 (524)
Oxazepam	6,386,352 (90)	5,695,946 (69)	4,963,544 (54)	4,015,017 (40)	4,160,395 (39)	3,986,512 (33)	3,644,592 (28)
Temazepam	37,064,285 (522)	40,487,830 (494)	42,890,739 (464)	39,642,909 (395)	35,112,223 (327)	31,928,995 (268)	29,138,759 (220)
Triazolam	2,550,427 (36)	4,429,516 (54)	4,738,528 (51)	4,429,364 (44)	5,495,847 (51)	6,567,877 (55)	6,320,590 (48)
Zolpidem	115,313,227 (1623)	102,107,687 (1246)	104,330,138 (1128)	95,799,044 (956)	78,435,056 (729)	73,375,604 (616)	70,525,182 (533)
Total^c^	487,828,377 (6866)	449,009,812 (5477)	397,092,128 (4292)	407,344,414 (4063)	381,182,739 (3545)	382,989,785 (3214)	366,881,546 (2772)
Antipsychotics							
Amoxapine	499,153 (7)	588,506 (7)	573,427 (6)	526,986 (5)	487,043 (5)	163,355 (1)	219,167 (2)
Aripiprazole	590,466,994 (8310)	732,163,777 (8932)	699,936,189 (7564)	379,335,492 (3784)	219,441,554 (2041)	182,150,245 (1529)	173,416,656 (1310)
Asenapine	9,728,156 (137)	111,213,935 (137)	13,569,011 (147)	16,873,815 (168)	19,123,233 (178)	21,280,694 (179)	21,756,410 (164)
Chlorpromazine	7,131,820 (100)	9,795,297 (119)	21,228,046 (229)	24,273,182 (242)	26,012,221 (242)	26,187,645 (220)	24,511,683 (185)
Clozapine	12,610,319 (177)	13,795,766 (168)	15,314,388 (166)	16,249,377 (162)	16,369,132 (152)	16,936,993 (142)	17,830,752 (135)
Haloperidol	6,769,277 (95)	10,944,735 (134)	11,059,976 (120)	10,376,258 (104)	9,768,919 (91)	9,210,830 (77)	6,922,736 (52)
Iloperidone	5,126,448 (72)	6,591,321 (80)	8,408,450 (91)	8,874,364 (89)	9,365,596 (87)	11,108,384 (93)	12,382,946 (94)
Loxapine	1,150,830 (16)	1,083,650 (13)	1,093,348 (12)	1,114,192 (11)	1,178,207 (11)	1,269,508 (11)	1,299,262 (10)
Olanzapine	171,954,481 (2420)	121,139,053 (1478)	68,793,400 (743)	53,922,986 (538)	53,003,088 (493)	55,484,734 (466)	57,189,092 (432)
Paliperidone	27,481,223 (387)	36,565,417 (446)	43,484,885 (470)	41,845,333 (417)	42,856,874 (399)	46,104,999 (387)	49,418,006 (373)
Quetiapine	329,435,654 (4637)	287,746,496 (3510)	239,227,530 (2585)	225,727,973 (2252)	164,615,907 (1531)	121,342,340 (1018)	124,077,930 (937)
Risperidone	64,862,457 (913)	50,992,510 (622)	42,461,823 (459)	34,760,864 (347)	33,501,159 (312)	35,111,661 (295)	36,775,415 (278)
Total^c^	1,227,216,812 (17,272)	1,282,620,463 (15,647)	1,165,150,475 (12,592)	813,880,822 (8119)	595,722,934 (5540)	526,351,388 (4417)	525,800,055 (3973)

^a^Total may be more or less than sum of columns due to rounding ^b^Dollars spent on PPI, benzodiazepine, or antipsychotics per $1,000,000 in drug costs in parentheses ^c^Per Cochran-Armitage trend test, *p*-value was < 0.0001 for all class level yearly trends

The number of PPI prescribers in each specialty group are in Table 4. The proportion of providers who prescribed PPIs decreased from 27.4% in 2013 to 25.7% in 2019 (*p* < 0.0001). Overall, general practitioners and mid-level practitioners had the highest number of providers prescribing a PPI. However, gastroenterology had the highest percentage (~ 93%) of providers prescribing a PPI all seven years. Table 5 shows number of standardized 30-day PPI claims from each specialty for beneficiaries over 65 years. Claims per 1000 are reported at the provider level due to lower counts compared to the overall counts because of suppression of counts < 11. The percent of PPI prescribers having all missing values for their standardized 30-day PPI claims increased from 25% in 2013 to 31% in 2019.The number of PPI claims per 1000 remained fairly steady across all seven years for all specialties, and the proportion of PPI claims to all claims had a significant decreasing trend (30.4 to 27.7 per 1000, *p* < 0.0001). Gastroenterologists prescribed 313.9 to 325.2 PPIs per 1000 standardized 30-day claims for beneficiaries over 65 years. General practitioners had the second highest PPI claim rate (31.9–35.1 per 1000) and specialists the lowest (6.1–9.5 per 1000). Table 6 shows PPI prescribers in each region. There were several regional differences. South had the highest number of PPI prescribers for beneficiaries over 65 years, but prescribers in Other region had the highest percentage prescribing a PPI. The South also had the highest number of standardized 30-day PPI claims for beneficiaries over 65 years and the highest number of PPI claims per 1000 claims (29.9–34.0). The West had the fewest PPI claims per 1000 claims (21.8–26.4). Table 7 shows the number of standardized 30-day claims for beneficiaries over 65 years for the regions.

**Table 4:** Number of specialists who prescribed a PPI, benzodiazepine, or antipsychotic each year^a^^,b^

Specialty	2013	2014	2015	2016	2017	2018	2019
PPI							
Gastroenterologists	11,739 (92.5%)	11,983 (92.8%)	12,150 (92.8%)	12,362 (92.6%)	12,582 (92.6%)	12,778 (92.3%)	12,988 (92.7%)
General Practitioners	159,412 (62.6%)	161,208 (63.4%)	162,412 (63.1%)	161,852 (62.4%)	161,821 (61.8%)	161,125 (60.9%)	160,972 (60.4%)
Specialists	54,827 (17.5%)	55,320 (17.6%)	54,259 (17.1%)	52,641 (16.5%)	51,018 (15.9%)	48,767 (15.1%)	46,854 (14.4%)
Surgeons	5572 (7.5%)	5593 (7.5%)	5496 (7.3%)	5289 (7.1%)	5392 (7.2%)	5207 (7.0%)	5158 (6.9%)
Mid-level Practitioners	55,398 (14.1%)	61,540 (14.8%)	68,336 (15.6%)	74,788 (16.1%)	80,947 (16.5%)	86,126 (16.3%)	92,616 (16.6%)
Alternative	34 (1.9%)	38 (2.8%)	32 (2.8%)	34 (2.9%)	28 (2.3%)	41 (3.1%)	26 (1.9%)
Total^c^	287,037 (27.4%)	295,682 (27.6%)	302,685 (27.5%)	306,966 (27.1%)	311,788 (26.8%)	314,044 (26.1%)	318,614 (25.7%)
Benzodiazepines							
Psychiatrists	25,849 (61.6%)	26,184 (62.4%)	26,069 (61.9%)	25,803 (61.4%)	25,436 (60.4%)	24,767 (58.7%)	24,277 (57.2%)
Sleep Medicine	68 (64.2%)	120 (64.9%)	163 (66.5%)	197 (62.5%)	227 (58.8%)	269 (61.4%)	306 (61.6%)
General Practitioners	131,531 (51.7%)	130,375 (51.3%)	129,094 (50.2%)	126,712 (48.9%)	124,210 (47.4%)	121,037 (45.8%)	117,819 (44.2%)
Specialists	51,493 (18.2%)	50,539 (17.7%)	48,813 (17.0%)	46,828 (16.2%)	45,082 (15.5%)	42,201 (14.3%)	39,515 (13.3%)
Surgeons	5397 (7.3%)	5251 (7.0%)	4778 (6.4%)	4609 (6.2%)	4512 (6.0%)	4042 (5.4%)	3632 (4.9%)
Mid-level Practitioners	42,804 (10.9%)	46,969 (11.3%)	51,411 (11.7%)	55,650 (12.0%)	60,194 (12.3%)	64,487 (12.2%)	68,868 (12.3%)
Alternative	53 (3.0%)	62 (4.6%)	61 (5.3%)	62 (5.3%)	71 (5.8%)	72 (5.4%)	66 (4.7%)
Total^c^	257,195 (24.5%)	259,500 (24.2%)	260,389 (23.6%)	259,861 (23.0%)	259,732 (22.3%)	256,875 (21.3%)	254,483 (20.5%)
Antipsychotics							
Psychiatrists	26,820 (63.9%)	26,881 (64.1%)	26,613 (63.2%)	26,875 (64.0%)	26,727 (63.4%)	26,282 (62.2%)	26,097 (61.5%)
Sleep Medicine	6 (5.6%)	11 (5.9%)	16 (6.5%)	16 (5.1%)	24 (6.2%)	27 (6.2%)	36 (7.2%)
General Practitioners	76,230 (29.9%)	76,679 (30.2%)	75,516 (29.3%)	76,617 (29.5%)	75,645 (28.9%)	74,276 (28.1%)	72,319 (27.1%)
Specialists	11,856 (4.2%)	11,766 (4.1%)	11,420 (4.0%)	11,468 (4.0%)	11,109 (3.8%)	11,064 (3.8%)	10,722 (3.6%)
Surgeons	518 (0.7%)	501 (0.7%)	416 (0.6%)	401 (0.5%)	350 (0.5%)	289 (0.4%)	293 (0.4%)
Mid-level Practitioners	20,596 (5.2%)	23,139 (5.6%)	25,642 (5.8%)	29,855 (6.4%)	33,345 (6.8%)	37,282 (7.1%)	41,566 (7.4%)
Alternative	22 (1.2%)	15 (1.1%)	13 (1.1%)	13 (1.1%)	12 (1.0%)	21 (1.6%)	12 (0.9%)
Total^c^	136,048 (13.0%)	138,992 (13.0%)	139,636 (12.7%)	145,245 (12.8%)	147,212 (12.7%)	149,241 (12.4%)	151,045 (12.2%)

^a^Percentage of all providers who prescribed a PPI, benzodiazepine, or antipsychotic in parentheses ^b^Denominators are not shown because they are specific to year and specialty, which are different for PPIs vs BZRA/APs ^c^Per Cochran-Armitage trend test, *p*-value was < 0.0001 for all class level yearly trends

**Table 5:** Number of standardized 30-day fills of PPIs, benzodiazepines, or antipsychotics for patients over 65 years per specialty^a^^,b^

Specialty	2013	2014	2015	2016	2017	2018	2019
PPI							
Gastroenterologists	3,809,834 (317.0)	4,129,780 (322.6)	4,310,201 (323.0)	4,436,205 (316.0)	4,635,020 (313.9)	4,926,534 (315.5)	5,313,923 (325.2)
General Practitioners	35,600,347 (34.2)	38,426,638 (35.1)	39,671,508 (35.0)	40,235,945 (34.1)	40,498,511 (33.3)	40,460,395 (32.3)	41,045,190 (31.9)
Specialists	2,898,758 (9.5)	3,024,364 (9.3)	2,973,763 (8.8)	2,886,237 (8.0)	2,758,802 (7.3)	2,620,119 (6.6)	2,560,219 (6.1)
Surgeons	234,553 (16.9)	233,795 (16.9)	232,863 (17.1)	224,062 (16.5)	231,727 (17.1)	223,396 (16.8)	228,458 (17.2)
Mid-level Practitioners	3,324,054 (25.0)	4,118,378 (26.2)	4,861,636 (26.3)	5,579,009 (25.8)	6,360,759 (25.2)	7,261,332 (24.7)	8,336,150 (24.5)
Alternative	962 (11.3)	583 (7.7)	578 (8.1)	680 (8.5)	740 (8.1)	1041 (9.7)	843 (7.3)
Total^c^	45,868,508 (30.4)	49,933,537 (31.1)	52,050,549 (30.9)	53,362,138 (29.9)	54,485,559 (29.0)	55,492,818 (28.1)	57,484,783 (27.7)
Benzodiazepines							
Psychiatrists	2,449,554 (142.7)	2,714,820 (145.3)	2,854,814 (144.8)	3,010,030 (142.7)	3,082,937 (137.9)	3,129,415 (133.2)	3,125,584 (126.8)
Sleep Medicine	2992 (63.2)	6554 (82.6)	8236 (69.8)	9682 (64.0)	12,294 (65.5)	13,472 (66.2)	15,070.1 (60.6)
General Practitioners	17,974,008 (17.2)	18,472,821 (16.9)	18,113,106 (16.0)	17,769,214 (15.1)	17,284,683 (14.2)	16,440,117 (13.1)	15,268,445 (11.9)
Specialists	2,019,714 (6.7)	2,054,486 (6.4)	1,934,717 (5.8)	1,876,413 (5.3)	1,802,349 (4.9)	1,708,778 (4.4)	1,560,540 (3.8)
Surgeons	105,522 (7.6)	100,279 (7.2)	91,213 (6.7)	81,479 (6.0)	74,245 (5.5)	62,284 (4.7)	55,910 (4.2)
Mid-level Practitioners	1,478,593 (11.1)	1,757,280 (11.2)	2,010,916 (10.9)	2,247,605 (10.4)	2,481,210 (9.8)	2,793,525 (9.5)	3,051,384 (9.0)
Alternative	549 (6.5)	540 (7.1)	424 (5.9)	577 (7.2)	794 (8.7)	831 (7.8)	833 (7.2)
Total^c^	24,030,931 (15.9)	25,106,780 (15.7)	25,013,425 (14.8)	24,995,000 (14.0)	24,738,511 (13.2)	24,148,423 (12.2)	23,077,765 (11.1)
Antipsychotics							
Psychiatrists	1,984,676 (115.6)	2,139,090 (114.7)	2,183,912 (110.8)	2,400,792 (113.8)	2,534,682 (113.4)	2,665,446 (113.4)	2,779,925 (112.8)
Sleep Medicine	111 (2.3)	318 (4.0)	431 (3.7)	746 (4.9)	784 (4.2)	677 (3.3)	914 (3.7)
General Practitioners	4,016,517 (3.9)	670,946 (4.3)	3,755,552 (3.3)	3,775,936 (3.2)	3,736,693 (3.1)	3,728,373 (3.0)	3,622,783 (2.8)
Specialists	355,425 (1.2)	367,238 (1.2)	357,839 (1.0)	379,348 (1.1)	375,167 (1.0)	396,176 (1.0)	401,214 (1.0)
Surgeons	21,892 (1.6)	20,283 (1.5)	16,967 (1.2)	13,896 (1.0)	12,528 (0.9)	10,848 (0.8)	10,012 (1.0)
Mid-level Practitioners	540,960 (4.1)	670,946 (4.3)	786,836 (4.3)	993,415 (4.6)	1,205,733 (4.8)	1,475,046 (5.0)	1,764,213 (5.2)
Alternative	87 (1.0)	116 (1.5)	105 (1.5)	171 (2.1)	90 (1.0)	191 (1.8)	91 (0.8)
Total^c^	6,919,668 (4.6)	7,172,717 (4.5)	7,101,642 (4.2)	7,564,302 (4.2)	7,865,678 (4.2)	8,276,758 (4.2)	8,579,151 (4.1)

^a^Number of standardized 30-day fills per 1000 standardized 30-day fills in parentheses ^b^Denominators are not shown because they are specific to year and specialty, which are different for PPIs vs BZRA/APs ^c^Per Cochran-Armitage trend test, *p*-value was < 0.0001 for all class level yearly trends

**Table 6:** Number of prescribers who prescribed a PPI, benzodiazepine, or antipsychotic each year in each region^a^^,b^

Region	2013	2014	2015	2016	2017	2018	2019
PPI							
Northeast	59,325 (25.2%)	60,795 (25.5%)	61,680 (25.2%)	62,623 (25.0%)	63,275 (24.8%)	63,556 (24.4%)	64,328 (24.2%)
Midwest	63,686 (27.7%)	65,767 (27.9%)	67,596 (28.0%)	68,715 (27.7%)	70,017 (27.4%)	70,679 (27.0%)	71,882 (26.8%)
South	99,871 (28.7%)	103,081 (28.8%)	105,643 (28.6%)	107,149 (28.1%)	109,733 (27.9%)	110,784 (27.0%)	112,586 (26.5%)
West	59,878 (26.7%)	61,714 (27.0%)	63,529 (27.0%)	64,371 (26.7%)	64,741 (26.1%)	65,071 (24.9%)	65,841 (24.3%)
Other	4277 (37.6%)	4325 (37.8%)	4237 (38.1%)	4108 (37.5%)	4022 (36.9%)	3954 (36.0%)	3977 (36.0%)
Total	287,037 (27.3%)	295,682 (27.6%)	302,685 (27.5%)	306,966 (27.1%)	311,788 (26.8%)	314,044 (26.1%)	318,614 (25.7%)
Benzodiazepines							
Northeast	55,291 (23.5%)	55,371 (23.2%)	55,223 (22.6%)	55,083 (22.0%)	54,504 (21.4%)	54,048 (20.8%)	53,801 (20.3%)
Midwest	55,561 (24.1%)	56,191 (23.8%)	56,673 (23.5%)	56,689 (22.8%)	56,330 (22.1%)	55,690 (21.3%)	55,224 (20.6%)
South	87,076 (25.0%)	88,249 (24.6%)	88,871 (24.1%)	88,843 (23.3%)	90,015 (22.9%)	89,487 (21.8%)	89,347 (21.0%)
West	55,580 (24.8%)	56,050 (24.5%)	56,132 (23.8%)	55,833 (23.1%)	55,465 (22.4%)	54,258 (20.8%)	52,835 (19.5%)
Other	3687 (32.4%)	3639 (31.8%)	3490 (31.4%)	3413 (31.2%)	3418 (31.4%)	3392 (30.9%)	3276 (29.7%)
Total	257,195 (24.5%)	259,500 (24.2%)	260,389 (23.6%)	259,861 (23.0%)	259,732 (22.3%)	256,875 (21.3%)	254,483 (20.5%)
Antipsychotics							
Northeast	29,495 (12.5%)	29,931 (12.5%)	29,847 (12.2%)	31,116 (12.4%)	31,536 (12.4%)	31,780 (12.2%)	31,807 (12.0%)
Midwest	30,866 (13.4%)	31,280 (13.3%)	31,470 (13.0%)	32,633 (13.1%)	33,133 (13.0%)	33,567 (12.8%)	33,767 (12.6%)
South	45,246 (13.0%)	46,548 (13.0%)	47,062 (12.7%)	49,002 (12.9%)	49,929 (12.7%)	50,922 (12.4%)	52,085 (12.3%)
West	28,609 (12.8%)	29,342 (12.8%)	29,364 (12.5%)	30,625 (12.7%)	30,735 (12.4%)	31,086 (11.9%)	31,568 (11.7%)
Other	1832 (16.1%)	1891 (16.5%)	1893 (17.0%)	1869 (17.1%)	1879 (17.3%)	1886 (17.2%)	1818 (16.5%)
Total	136,048 (13.0%)	138,992 (13.0%)	139,636 (12.7%)	145,245 (12.8%)	147,212 (12.7%)	149,241 (12.4%)	151,045 (12.2%)

^a^Percentage of all providers who prescribed a PPI, benzodiazepine, or antipsychotic in parentheses ^b^Denominators are not shown because they are specific to year and region

**Table 7:** Number of standardized 30-day fills of PPIs, benzodiazepines, or antipsychotics for patients over 65 years per region^a^^,b^

Region	2013	2014	2015	2016	2017	2018	2019
PPI							
Northeast	8,768,992 (30.4)	9,465,198 (31.1)	9,795,065 (30.8)	10,081,212 (29.8)	10,273,812 (29.2)	10,501,229 (28.3)	10,853,244 (27.9)
Midwest	10,254,207 (29.6)	11,256,768 (30.6)	11,902,161 (30.9)	12,323,442 (30.4)	12,646,944 (29.8)	12,943,790 (29.1)	13,432,506 (29.0)
South	18,493,221 (33.3)	20,284,181 (34.0)	21,046,315 (33.4)	21,541,143 (32.1)	22,263,931 (31.4)	22,743,638 (30.3)	23,655,972 (29.9)
West	7,765,420 (26.4)	8,271,129 (26.5)	8,574,494 (26.2)	8,682,084 (25.1)	8,595,189 (23.7)	8,579,843 (22.4)	8,748,059 (21.8)
Other	586,668 (27.8)	656,261 (28.7)	732,514 (30.1)	734,257 (29.1)	705,684 (26.9)	724,319 (26.0)	795,003 (26.3)
Total	45,868,508 (30.4)	49,933,537 (31.1)	52,050,549 (30.9)	53,362,138 (29.9)	54,485,559 (29.0)	55,492,818 (28.1)	57,484,783 (27.7)
Benzodiazepines							
Northeast	3,969,607 (13.7)	4,089,129 (13.5)	4,105,101 (12.9)	4,203,292 (12.4)	4,170,618 (11.8)	4,207,875 (11.4)	4,196,429 (10.8)
Midwest	4,743,611 (13.7)	4,994,795 (13.6)	5,069,499 (13.2)	5,051,528 (12.4)	4,955,922 (11.7)	4,791,099 (10.8)	4,551,203 (9.8)
South	10,881,430 (19.6)	11,417,185 (19.1)	11,308,658.5 (17.9)	11,235,543 (16.8)	11,154,306 (15.7)	10,763,750 (14.3)	10,146,007 (12.8)
West	3,936,237 (13.4)	4,048,233 (13.0)	3,943,805 (12.1)	3,870,739 (11.2)	3,780,459 (10.4)	3,665,271 (9.6)	3,464,867 (8.6)
Other	500,045 (23.7)	557,438 (24.4)	586,362 (24.1)	633,898 (25.1)	677,208 (25.9)	720,428 (25.9)	719,259 (23.8)
Total	24,030,931 (15.9)	25,106,780 (15.7)	25,013,425 (14.8)	24,995,000 (14.0)	24,738,511 (13.2)	24,148,423 (12.2)	23,077,765 (11.1)
Antipsychotics							
Northeast	1,593,013 (5.5)	1,621,897 (5.3)	1,606,992 (5.1)	1,702,868 (5.0)	1,737,370 (4.9)	1,819,882 (4.9)	1,868,958 (4.8)
Midwest	1,610,290 (4.6)	1,649,822 (4.5)	1,622,308 (4.2)	1,727,398 (4.3)	1,781,193 (4.2)	1,863,025 (4.2)	1,914,522 (4.1)
South	2,455,290 (4.4)	2,567,727 (4.3)	2,532,400 (4.0)	2,685,679 (4.0)	2,837,491 (4.0)	2,985,785 (4.0)	3,105,362 (3.9)
West	1,159,470 (3.9)	1,213,165 (3.9)	1,214,101 (3.7)	1,314,064 (3.8)	1,374,306 (3.8)	1,462,838 (3.8)	1,532,942 (3.8)
Other	101,605 (4.8)	120,107 (5.3)	125,841 (5.2)	134,294 (5.3)	135,319 (5.2)	145,227 (5.2)	157,368 (5.2)
Total	6,919,668 (4.6)	7,172,717 (4.5)	7,101,642 (4.2)	7,564,302 (4.2)	7,865,678 (4.2)	8,276,758 (4.2)	8,579,151 (4.1)

^a^Number of standardized 30-day PPI, benzodiazepine, or antipsychotic fills per 1000 standardized 30-day fills for patients over 65 years in parentheses ^b^Denominators are not shown because they are specific to year and region

### Antipsychotics

The use of APs by beneficiaries was low and slightly decreasing from 2013 to 2019 (4.6% to 4.1%, *p* < 0.0001). Less than 5% of beneficiaries used an AP with quetiapine being the most common at 1.9% each year. Table 1 shows rates of beneficiary AP use. Although rates of beneficiary use were low, standardized 30-day claims per 1,000,000 were relatively high, but trending downward, ranging from 6,316.1 to 6,797.3 (*p* < 0.0001). Total standardized 30-day AP claims increased by over 3 million from 2013 to 2019. Quetiapine and risperidone together accounted for over half of these claims. Standardized 30-day claims of other APs are in Table 2.

The aggregate costs of APs are in Table 3. Total costs of APs more than halved from over $1.2 billion in 2013 to over $500 million in 2019, and costs per $1,000,000 were more than quartered from $17,272 to $3973 (*p* < 0.0001). Aripiprazole had the greatest decrease in costs from nearly $600 million ($8,310 per $1,000,000) in 2013 to under $200 million ($1310 per $1,000,000) in 2019. This decrease in cost was likely due to generic approval for aripiprazole in 2015.

The percent of providers prescribing APs significantly decreased across seven years (13.0% to 12.2%, *p* < 0.0001). Psychiatrists had the highest prescribing rate at just over 60%, and general practitioners were the second highest prescribers at or under 30%. Each provider type prescribed 5 or less APs for every 1,000 claims, except psychiatrists who had 110.8–115.6 standardized 30-day claims per 1,000. Claims per 1000 are reported at the provider level due to lower counts compared to the overall counts because of suppression of counts < 11. The percent of AP prescribers having all missing values for their standardized 30-day AP claims increased from 17.6% in 2013 to 24.7% in 2019. Provider type prescribing rates and standardized 30-day claims for APs are in Tables 5 and 6, respectively. Other region had the highest AP prescribing rate (16–17%). All other regions had similar prescribing rates at 11–13%. Northwest and Other region had around 5 standardized 30-day claims per 1,000 versus 4 for the others. The total claim rates significantly decreased over the seven years (4.6 to 4.1, *p* < 0.0001). Prescriber and claim rates were stable for all regions. Regional prescribing and claim rates for APs are in Tables 6 and 7, respectively.

### Benzodiazepines

Table 1 shows rates of BZRA use in Medicare beneficiaries. The overall rate of BZRA use declined from 2013 to 2019 with 25.6% using at least one in 2013 and 17.5% in 2019 (*p* < 0.0001). Alprazolam, lorazepam, and zolpidem each had over one million beneficiaries each year and were the top BZRAs used. However, the standardized 30-day claims per 1,000,000 for alprazolam (5,934.7 in 2013 to 4775.0 in 2019) AND lorazepam slightly decreased (5,087.5 in 2013 to 3526.5 in 2019), and zolpidem use was halved (6,295.6 in 2013 to 3107.4 in 2019). Overall, the proportion of standardized claims for BZRAs have decreased from 24,766.4 to 17,024.1 (*p* < 0.0001).

Overall, aggregate costs for BZRAs decreased. Total overall costs per $1,000,000 decreased from $6,866 in 2013 to $2772 in 2019 (*p* < 0.0001). Eszopiclone and zolpidem had the largest decrease in costs per $1,000,000. Chlordiazepoxide and clorazepate more than doubled in costs per $1,000,000 from 2013 to 2017, although their costs remained low. Table 3 contains other costs for BZRAs.

The percentage of providers who prescribed a BZRA decreased from 24.5% in 2013 to 20.5% in 2019 (*p* < 0.0001). Sleep medicine, psychiatrists, and general practitioners were the top prescribers with 58.8–66.5%, 57.2–62.4%, and 44.2–51.7% of these providers prescribing BZRAs, respectively. Other prescriber rates are in Table 4. The number of standardized 30-day claims from providers were stable overall across the seven-year period at 23–25 million. Standardized 30-day claims per 1,000 claims slightly decreased from 15.9 to 11.1 (*p* < 0.0001). Psychiatrists had highest standardized 30-day BZRA claims per 1,000 over the seven years, ranging from 126.8 to 145.3. Table 5 shows standardized BZRA claims per provider type. Claims per 1000 are reported at the provider level due to lower counts compared to the overall counts because of suppression of counts < 11. The percent of BZRA prescribers having all missing values for their standardized 30-day BZRA claims ranged from 21.4% to 22.8%. Other region had about 30% of providers prescribing a BZRA and also had over 20 standardized 30-day claims per 1,000 each year. BZRA prescriber rates and standardized 30-day claims per region are in Tables 6 and 7, respectively.

Sensitivity analyses of the above trend analyses using logistic regression were all significant at *p* < 0.0001 using both the Wald test and likelihood ratio test.

## Discussion

Overall, prescribers of all three drug classes decreased or remained stable over all seven years in each specialty except for mid-level practitioners and alternative medicine practitioners, for whom increasing numbers of providers wrote prescriptions for the three classes. Likewise, standardized 30-day claims for all drug classes per specialty decreased or remained stable except alternative medicine with BZRAs and sleep medicine and mid-level practitioners with APs. Regionally, prescriber rates of all three drug classes decreased or remained stable with the exception of Other region with increasing prescribing of APs. Similarly, standardized 30-day claims per 1,000 were decreasing or stable across regions except the other region with BZRAs.

The overall prevalence of PPI prescriptions across all seven years was around 30%. This is slightly higher than the 27% prevalence found among older adults residing in nursing homes in 2004 [35]. Being a Medicare beneficiary was one of the significant predictors of inappropriate PPI use among the nursing home residents [15] and this higher prevalence of PPI prescriptions is possibly due to the implementation of Medicare Part D, offering increased access to these medications. One time-trend analysis of pharmacy claims data found a 37% increase in PPI prescriptions among seniors after Medicare Part D was implemented [36]. This may be due to seniors switching from over-the-counter to prescription PPIs [36]. PPI prescriptions did not increase among dual eligible beneficiaries after the implementation of Medicare Part D even though the copayments decreased [37].

Although total number of PPI claims have increased from 2013 to 2019, the total cost of PPIs has decreased. The contributing factors for this decrease may be the first generic version of esomeprazole being approved in early 2015 [38] and over-the-counter esomeprazole becoming available in 2014 [39]. The aggregate cost of esomeprazole has decreased by almost $1.5 billion from 2015 to 2019. In 2013, esomeprazole was the second top selling drug in Medicare Part D claims for beneficiaries over 65 years with $1.833 billion in aggregate costs. In 2015, it was the eighth top drug with $1.429 billion in aggregate costs and fell out of the top 10 in 2016, although the decrease in number of claims may also contribute to this decrease in costs. Dexlansoprazole claims have slightly increased, and its costs have doubled from 2013 to 2019. Generic dexlansoprazole 60 mg was not approved until 2017 [40]. Pantoprazole claims have nearly doubled, and with that, costs have increased. This may be due to pantoprazole being one of the preferred PPIs to use in patients on clopidogrel because it is less likely to inhibit CYP2C19, which metabolizes clopidogrel into its active form [41].

Over 90% of gastroenterologists have prescribed a PPI to patients over 65 years from 2013–19. Gastroenterologists also out-prescribed PPIs almost ten times the rate of other specialties. Although the number of PPI prescriptions from general practitioners has increased, the number of PPI claims per 1,000 has remained steady. Specialists slightly decreased their number of PPI prescriptions and had a decreased number of providers prescribing a PPI. This is similar to the increased PPI prescriptions found among family practice prescribers and gastroenterologists and the decrease among otolarynologists from 2013–16 [42].

Alprazolam, lorazepam, and zolpidem were the most used BZRAs among beneficiaries. However, while alprazolam use and claims remained stable, lorazepam and zolpidem users and standardized 30-day claims decreased. Psychiatrists were one of the top prescribers of BZRAs at around 60%, and overall provider prescribing rate was under 25%. However, another study using 2016 Medicare Part D data found over 80% of psychiatrists prescribed a BZRA, and 26% of all providers prescribed a BZRA [43]. This difference between results may be due to our limiting analysis to beneficiaries 65 years and older. This is supported by a study that found adults between 65 and 80 years who were using BZRAs were less likely to get it from a psychiatrist compared to other age groups [29]. Determining and targeting top alprazolam prescribers could be the next step for deprescribing BZRAs in adults over 65 years.

The sharp decrease in zolpidem standardized 30-day claims from 2014 could be due to its addition to the 2012 Beers Criteria and implementation of increased utilization management for prescriptions [44]. The proportion of patients receiving a z-sleep drug (eszopiclone or zolpidem) was higher in this study compared to 1.5% rate found in a Federally Qualified Health Center from 2016–17 [45]. Although some generic versions of generic eszopiclone were approved in 2011, the sharp decrease in aggregate costs for eszopiclone since 2014 could possibly be explained by the majority being approved in 2013 and later [39]. Sleep medicine physicians were also among the top prescribers for BZRAs. This could be a potential group to target for deprescribing efforts.

The decrease in costs for APs is probably driven by aripiprazole going generic in 2015 [46] and quetiapine extended release going off patent in 2017 [47]. In 2006 aggregate costs for antipsychotics were nearly $700 million for Medicare beneficiaries [48].

The high prevalence of PPIs prescriptions indicates an opportunity to introduce deprescribing protocol as part of daily practice. Physician specialties that prescribe more PPIs, APs and BZRAs may be potentially targeted for deprescribing interventions. Several studies have looked at the effectiveness of deprescribing implementation techniques in different settings. Avraham et al. established and implemented a tapering protocol to mitigate overuse of PPIs in Nursing Homes and found that 90% of the selected residents achieved cessation [49]. Odenthal et al. carried out an intervention in order to improve the rate of discontinuation of PPIs in primary care setting [50]. The study resulted in the intervention having a higher successful rate of deprescribing of PPIs than other previous studies in primary care setting [50]. This study shows that a combination of pharmacist patient education, written tapering schedule, symptom action plan and follow up with the help of appropriate guidelines can lead to successful deprescribing of medications [50]. Yet, it is unclear whether such deprescribing interventions lead to better clinical outcomes [51].

Similarly, deprescribing interventions have been developed for anti-psychotics and benzodiazepines. Pottie and colleagues developed an evidence-based guideline for safely tapering and stopping the use of BZRAs [4]. This guideline recommends older adults who receive benzodiazepine should be offered deprescribing [4]. The deprescribing methodology recommended is a patient-centered, stepped down approach including a gradual dose reduction which is an integral part in the management of benzodiazepine use disorder [4]. Conn et al. recommends that older adults should be prescribed benzodiazepines only after patient education which includes knowledge about alternatives, benefits and risks related to their use [52]. Several interventions have been carried out for successfully discontinuing long-term use of benzodiazepine. Vicens and colleagues carried out interventions in primary care to enable patients to reduce or discontinue long-term benzodiazepine use. The intervention included either usual care, usual care with follow-up visits and usual care followed by written instructions [53]. Both follow-up interventions saw 45% of patients discontinue benzodiazepine suggesting a structured intervention with written gradual dose reduction can be effective in primary care [53]. Tannenbaum and colleagues carried out direct patient education to reduce inappropriate benzodiazepine prescriptions among older users [54]. The study resulted in 62% to initiate a conversation about benzodiazepine reduction with either a general practitioner and/or a pharmacist leading to 27% of participants to discontinue unnecessary benzodiazepines post intervention suggesting patient education can improve decision making regarding the inappropriate overuse of medications [54].

Farrell et al. recommends deprescribing antipsychotics for adults with behavioral and psychological symptoms of dementia [3]. Pan et al. carried out a systematic review and meta-analysis of randomized controlled studies for discontinuation of anti-psychotics in patients with dementia comparing severity and change of behavioral and psychological symptoms [55]. The meta-analysis showed that the group which discontinued antipsychotic use were not statistically significant in behavioral and psychological symptoms however, they showed lower mortality and higher early study termination rates during follow-up [55]. Education of antipsychotics for both healthcare professionals and patients may improve the discontinuation practice. Different interventions highlight the need for training and patient education about medication effectiveness and safety to enable older patients to initiate deprescribing of potentially inappropriate medications or prescriptions.

### Limitations

There are some limitations to the current work. First, some PPIs are available over the counter, which are not captured in these datasets. Thus, it is possible that the prevalence of PPI use is underestimated. Second, there is a possibility that beneficiaries received more than one type of drug in each class in a year and are therefore double counted. Duplication of drugs from same class is not common, and thus, we think that the prevalence of use of these medication classes may only be slightly overestimated. Third, beneficiaries could have received one or more drug prescriptions from multiple providers, which would also overestimate the prevalence of use. Fourth, if the beneficiary count is less than 11 for an observation, then the beneficiary count is removed from the dataset to protect patient data. Therefore, the total beneficiary count may be underestimated.

This was a descriptive exploratory study on three medication classes potentially used inappropriately in the geriatric Medicare population using publicly available datasets. These results are generalizable to the older adult Medicare Part D population, but they may not be an accurate reflection of US older adults who do not have Medicare Part D. Future work should focus on a more thorough analysis of these prescribing and cost trends using the Part D and Medicare data, allowing for more accurate estimation of beneficiaries using each class, and adjusting for important confounders. The dataset used in this study does not contain information on indication, and therefore, multiple appropriate uses for the three classes studied could not be captured. In addition, the dataset did not have any information on clinical outcomes. Outcomes associated with use of these drug classes can also be investigated with Medicare and Part D data, adding to the existing literature to identify areas to focus on dissemination and implementation of deprescribing interventions.

## Conclusion

This was a descriptive study on the general prevalence of prescriptions considered potentially inappropriate in the older adult Medicare population. About 30% of all Medicare Part D beneficiaries over 65 years of age used a PPI each year from 2013–19, costing an average of $2.4 billion each year. BZRAs users decreased from 25.6% to 17.5% from 2013–19, but costs only decreased a little over $100 million averaging $410 million per year. AP users were relatively stable at just over 4% of Medicare Part D beneficiaries from 2013–19, and costs decreased by more than 50% averaging $877 million each year. Despite the increase in number of PPI and AP claims, the cost of both have decreased over the years studied. The study also indicated notable differences in specialist prescribing and regional differences that may be targeted for dissemination and implementation of interventions for deprescribing of BZRAs and APs.

## Data Availability

The datasets used for this study are publicly available from Centers for Medicare & Medicaid Services at https://www.cms.gov/Research-Statistics-Data-and-Systems/Statistics-Trends-and-Reports/Medicare-Provider-Charge-Data/Part-D-Prescriber.

## Abbreviations

AP: Antipsychotic
BZRA: Benzodiazepine receptor agonist
PPI: Proton pump inhibitor
